# Equity of primary care service delivery for low income “sicker” adults across 10 OECD countries

**DOI:** 10.1186/s12939-018-0892-z

**Published:** 2018-12-12

**Authors:** Simone Dahrouge, William Hogg, Elizabeth Muggah, Ted Schrecker

**Affiliations:** 1 Department of Family Medicine and Scientist at the Bruyère Research Institute and the Institute of Clinical Evaluative Sciences, University of Ottawa, Ottawa, Canada; 2 Department of Family Medicine and Scientist at the Institut de Recherche de L’Hôpital Montfort and the Institute of Clinical Evaluative Sciences, University of Ottawa, Ottawa, Canada; 3 Department of Family Medicine, The University of Ottawa, Ottawa, Canada; 4 Institute of Health and Society, Newcastle University, Newcastle upon Tyne, UK

**Keywords:** Primary care, Household income, Health equity

## Abstract

**Background:**

Despite significant investments to support primary care internationally, income-based inequities in access to quality health care are present in many high-income countries. This study aims to determine whether low- and middle-income groups are more likely to report poor quality of primary care (PC) than high-income groups cross-nationally.

**Methods:**

The 2011 Commonwealth Fund Telephone Survey of Sicker Adults is a cross-sectional study across eleven countries. Respondents were recruited from randomly selected households. We used data from surveys conducted in Australia, Canada, France, Germany, the Netherlands, New Zealand, Norway, Sweden, the United Kingdom, and the United States. We identified all questions relating to primary care performance, and categorized these into five dimensions: 1) access to care, 2) coordination 3) patient-centered care, and 4) technical quality of care. We used logistic regression with low and middle-income as the comparison groups and high-income as the referent.

**Results:**

Fourteen thousand two hundred sixty-two respondents provided income data. Countries varied considerably in their extent of income disparity. Overall, 24.7% were categorized as low- and 13.9% as high-income. The odds of reporting poor *access to care* were higher for low- and middle-income than high-income respondents in Canada, New Zealand and the US. Similar results were found for Sweden and Norway on *coordination*; the opposite trend favoring the low- and middle-income groups was found in New Zealand, United Kingdom, and the United States. The odds of reporting poor *patient-centered care* were higher for low-income than high-income respondents in the Netherlands, Norway, and the US; in Australia, this was true for low- and middle-income respondents. On technical quality of care, the odds of reporting poor care were higher for the low- and middle-income comparisons in Canada and Norway; in Germany, the odds were higher for low-income respondents only. The odds of reporting poor technical quality of care were higher for high-income than low-income respondents in the Netherlands.

**Conclusion:**

Inequities in quality PC for low and middle income groups exist on at least one dimension in all countries, including some that in theory provide universal access. More research is needed to fully understand equity in the PC sector.

**Electronic supplementary material:**

The online version of this article (10.1186/s12939-018-0892-z) contains supplementary material, which is available to authorized users.

## Introduction

Equity is a valued aspect of most health care delivery systems. The International Society for Equity in Health (ISEqH) has defined equity as “the absence of potentially remediable, systematic differences in one or more aspects of health across socially, economically, demographically, or geographically defined population groups or subgroups” [1]. Evidence of health disparities related to income inequality has been shown consistently in many parts of the world [2–4]. More specifically, income-based inequities in access to quality health care have been demonstrated in many high-income countries, including some that in theory provide universal access to publicly funded care [5]. Primary care (PC) is the bedrock of a health care system. PC providers, usually a family physician or nurse practitioner, offer patient-centered care spanning the individual’s life trajectory and health needs [6]. They coordinate and facilitate access to health services across sectors. Deficiencies in that foundation can have serious repercussions. A health system’s orientation toward PC is related to better experience of care [7] and better health outcomes [4, 8]. Additionally, Starfield and colleagues demonstrated that health care systems oriented to PC are also associated with greater health equity [7, 9, 10].

The World Health Organization has advocated for strong PC sectors within health care systems, [11] and significant investments have been made to support PC internationally [12]. While past work has demonstrated the value of a greater health system orientation to PC, including enhanced system efficiency, better population health, and more equitable access to health services [8, 13], inequities within the PC sector itself remain largely unexamined. When access to PC is defined as the frequency of contact with the PC provider, several studies conducted in a setting where the costs of physician visits are covered by a universal insurer, found pro-poor or neutral effect of low socio-economic measures on access [14], while those conducted in a privatized settings found the opposite [15]. Studies assessing the equitable receipt of services within that sector are mixed. Studies showing equivalent or better care for vulnerable populations [16–18] relied predominantly on processes of care indicators carried out by the PC provider, while those revealing meaningful gaps, relied on measures of whether the individuals had undergone recommended tests, [19, 20] indicators that require an action by the individual.

Given the PC sector’s role in reducing gaps in the health of the population across sociodemographic strata, it may be informative to examine more broadly whether access to that sector across several dimensions, and within the same geo-political context to determine the fairness of that sector itself [21]. The Commonwealth Fund is a private non-profit organization based in the United States that routinely surveys patients and health care providers in OECD countries in order to measure health care performance across multiple developed nations. In 2011 they surveyed a large sample of “sicker” adults across eleven countries and captured detailed information on their experiences with the health care system [7]. Using patient survey data collected in 2011 across ten of these 11 Organization for Economic Cooperation and Development (OECD) countries by the Commonwealth Fund (Switzerland was omitted for reasons explained below), we conducted a cross-country comparison of the experience of low-income individuals across four dimensions of PC: 1) access to care, 2) coordination 3) patient-centered care, and 4) technical quality of care.

The goal of this study is to examine 1) the relationship between income level and the experience of quality primary health care across the four central dimensions of care in each country, and 2) whether equity of PC service varies across countries.

## Methods

### Sample

This study relied on respondents participating in the 2011 Commonwealth Fund Telephone Survey of Sicker Adults. Respondents were eligible if they 1) were in poor to fair health, 2) had received medical care for a chronic or serious illness, a major injury, or a disability in the previous year, 3) had been hospitalized in the past 2 years for reasons other than a complication-free childbirth, or 4) had undergone major surgery in the past 2 years.

### Survey design

The 2011 Commonwealth Fund Telephone Survey of Sicker Adults is a cross-sectional study across eleven countries: Australia, Canada, France, Germany, the Netherlands, New Zealand, Norway, Sweden, Switzerland, the United Kingdom, and the United States. Briefly, between March and June 2011, randomly selected households were contacted, and the resident who a) was over 18 years of age and b) had the most recent birthday was asked to respond to the questionnaire. Interviewers asked respondents a series of questions and noted their answers on a Likert scale. Details of the survey methodology are provided in Schoen et al. [7]. Country level response rates ranged from 16 to 42%. The minimum sample size required to detect an effect in each country was 750 respondents. However, several countries contributed additional funds to increase their sample size and allow additional power. The total number of participants ranged from 750 in New Zealand to 4804 in Sweden. A weight reflecting the population distribution of age, sex, level of education, and region was established in order to allow population-based estimates.

#### Respondent profile

The survey captured respondent household income, demographics (age, sex, immigration status, level of education), and health (self-reported measures of various chronic conditions) measures. Household income was captured in the survey using a six-option categorical variable for all countries except Switzerland and the United States. The options reflected values relative to that country’s respective median household income as follows: less than 50% of median, between 50 and 89% of median, between 90 and 109% median, between 110 and 149% median, between 150 and 200% median, and more than 200% median. In the United States, a five-item scale that reflected the country’s defined Federal Poverty Level was used [7]. Switzerland opted out of the income related question; as such, that country was excluded in this study. Each respondent was also asked to report the number of adults and children in their household (Additional file 1).

### Patient experience with PC

We identified all questions relating to PC performance, and mapped these according to previously defined dimension of care [22]. We identified five dimensions covered by the question surveys: 1) access to care, 2) continuity of care, 3) patient-centered care, 4) coordination, and 5) technical quality of care. However, because there was only a single question related to continuity of care, and a single question would not provide adequate coverage of that dimension, we dropped this dimension from our analyses. All other dimensions consisted of 3 to 6 questions. Likert scale questions were dichotomized into: acceptable and less than acceptable. For instance, in the original survey, the question “How often does your regular doctor or someone in your doctor’s practice help coordinate or arrange the care you receive from other doctors and places, such as make appointments?” could be answered 1) always, 2) often, 3) sometimes, or 4) rarely/never (Additional file 1). We classified the first two as acceptable, and the latter two as less than acceptable. If a respondent’s answer to at least one question within a dimension was ranked as less than acceptable, that respondent was coded as having received “poor” care on that dimension. Although this approach may be seen as introducing a negative bias in the results, it can also be interpreted as maximizing sensitivity to departures from the norm of equity [23].

### Analysis

Individuals who reported having a household income in the lowest income level were categorized as low-income; those with household income in the two highest income levels were categorized as high-income. All others were assigned to the middle-income category. We calculated the proportion of respondents who reported “poor” care for each income group. We used logistic regression to compare the responses of low- and middle-income respondents to those in the high-income category. Each of the four performance dimensions were, in turn, the dependent variables, while household income acted as the main independent variable, with the high-income level as a reference. The main analysis was unweighted and adjusted only for the number of individuals in the household to reflect available resources per person [24]. These analyses were repeated using population weight to produce an estimate reflecting the country’s population.

## Results

Of the 17,167 respondents, 14,262 (83.1%) provided income data (Table 1). Countries varied considerably in their extent of income disparity. Overall, 24.7% were categorized as low- (range: 13.2, 34.3%) and 13.9% as high- (range: 2.4, 21.4%) income. The United States had the largest spread in income, with 50.4% of individuals falling in either extreme income groups (low or high), while the United Kingdom had the narrowest spread (15.6%). There was an evident gradient relationship between income and several respondent characteristics (Table 2). Individuals with lower income had fewer people in the households, were older, had lower education, and reported worse health. They were also much more likely to be female. Individuals having not provided income information had a profile that resembled more closely that of individuals with low or medium income.

**Table 1:** Description of respondents by income group (Low, Medium, High)

Countries	Overall	Aus	Can	Fr	Ger	NT	NZ	NW	SW	UK	US
N	17,167	1,500	3,958	1,001	1,200	1,000	750	753	4,804	1,001	1,200
With income data (%)	83.1	82.8	80.9	87.4	71.5	74.4	85.3	90.7	87.7	83.1	81.0
# in the household (Mean, SD)											
Low	1.7 (1.1)	1.8 (1.0)	1.8 (1.1)	1.9 (1.3)	1.7 (1.0)	1.8 (1.2)	1.9 (1.3)	1.6 (0.8)	1.4 (0.8)	1.9 (1.5)	1.0 (1.5)
Medium	2.5 (1.3)	2.6 (1.4)	2.6 (1.4)	2.7 (1.4)	2.5 (1.3)	2.4 (1.4)	2.7 (1.5)	2.1 (1.0)	2.2 (1.2)	2.9 (1.2)	2.4 (1.4)
High	3.0 (1.4)	3.2 (1.1)	3.2 (1.4)	3.3 (1.4)	2.7 (1.3)	3.3 (1.6)	3.3 (1.4)	3.2 (1.4)	2.9 (1.3)	3.2 (1.2)	2.8 (1.9)
Unknown	2.2 (1.3)	2.2 (1.3)	2.3 (1.4)	2.1 (1.1)	2.4 (1.3)	2.4 (1.6)	2.4 (1.3)	1.9 (1.0)	1.9 (1.1)	2.6 (1.3)	2.1 (1.4)
Age (years) (mean years, SD)											
Low	63.4 (16.7)	65.7 (13.4)	61.8 (16.3)	57.1 (18)	60.3 (18.1)	62.7 (18)	63.9 (15.1)	65 (13.3)	67 (16.9)	61.6 (16.7)	63.4 (16.8)
Medium	55.4 (16.1)	54.3 ( 14.6)	53.2 (16.0)	51.7 (15.5)	52.9 (15.3)	58.1 (17.3)	53.2 (15.8)	60 (13.6)	57.8 (16.3)	49.6 (15.2)	61.0 (16.2)
High	50.5 (13.0)	48.6 (11.8)	48.3 (12.1)	51.7 (13.9)	49.4 (13.1)	51.3 (14.5)	49.9 (10.7)	51 (12.9)	51.5 (13.4)	51.7 (12.3)	54.5 (14.8)
Unknown	61.0 (18)	62.9 (16.7)	60.0 (17.3)	60.7 (19)	56.0 (18.0)	62.9 (18.7)	61.6 (15.5)	64.5 (18.0)	62.3 (18.4)	55.0 (19.4)	67.1 (16.6)
Female (%)											
Low	68.1	70.9	69.3	71.3	64.6	67.3	71.5	70.0	65.8	50.0	71.3
Medium	55.4	58.7	62.0	61.8	63.1	51.4	65.0	45.4	47.7	51.0	66.2
High	50.1	56.5	64.2	44.7	48.7	48.0	56.8	53.2	34.6	55.0	55.8
Unknown	69.4	74.4	66.9	70.6	71.3	83.2	70.0	71.4	62.2	66.3	72.4
College/University (%)											
Low	17.5	14.1	21.0	9.3	43.3	9.4	39.6	2.8	13.9	5.5	19.5
Medium	35.1	32.7	47.4	30.5	55.3	26.0	54.8	17.9	29.5	17.5	40.1
High	61.8	58.8	69.7	65.8	63.4	68.0	73.2	36.5	55.0	75.0	73.1
Unknown	28.4	24.3	39.3	14.6	31.6	15.0	51.5	8.7	23.1	23.0	31.0
Immigrant (%)											
Low	13.1	19.5	15.9	7.3	13.8	11.7	19.0	0.9	10.9	14.5	9.9
Medium	12.4	17.5	16.7	9.5	14.4	8.1	23.5	3.8	9.2	14.4	8.9
High	11.0	19.9	14.6	7.9	8.9	6.0	19.2	4.0	6.6	15.0	9.1
Unknown	14.8	21.7	20.7	8.8	16.5	8.6	21.8	4.3	10.4	15.4	5.4
Self-reported health > Good (%)											
Low	48.0	52.9	46.3	41.3	32.6	35.2	65.8	32.7	61.2	37.3	35.8
Medium	68.0	71.7	68.4	58.7	54.0	44.6	79.3	55.2	77.3	67.7	60.5
High	82.1	82.4	79.7	65.8	66.4	64.0	88.7	80.2	88.0	80.0	81.7
Unknown	59.1	62.6	61.1	39.7	52.6	40.2	67.3	43.5	71.6	61.5	56.4
# of Chronic Conditions^a^ (mean)											
Low	2.2 (1.5)	2.5 (1.4)	2.3 (1.6)	1.9 (1.5)	2.4 (1.6)	1.8 (1.4)	2.1 (1.4)	2.2 (1.5)	1.7 (1.3)	2.3 (1.7)	2.9 (1.7)
Medium	1.4 (1.3)	1.7 (1.5)	1.5 (1.4)	1.4 (1.3)	1.6 (1.4)	1.4 (1.3)	1.3 (1.2)	1.6 (1.3)	1.2 (1.2)	1.3 (1.4)	2.2 (1.6)
High	1 1 (1.2)	1.1 (1.2)	1.2 (1.3)	1 (1.1)	1.2 (1.1)	1.1 (1.1)	0.9 (1)	1 (0.9)	0.8 (1)	1.2 (1.5)	1.5 (1.3)
Unknown	1.6 (1.4)	1.9 (1.5)	1.6 (1.4)	1.7 (1.4)	1.7 (1.4)	1.6 (1.3)	1.7 (1.4)	1.6 (1.4)	1.1 (1.2)	1.6 (1.6)	2.2 (1.6)

Aus = Australia, Can = Canada, Fr = France, Ger = Germany, NT = Netherlands, NZ = New Zealand, NW = Norway, SW = Sweden, UK = United States

^a^The chronic conditions assessed were: Hypertension, Heart disease, Diabetes, Joint pain/Arthritis, Asthma/COPD/any other chronic lung problem, Depression/Anxiety/other mental health problem, Cancer, Chronic back pain

**Fig. 1:**
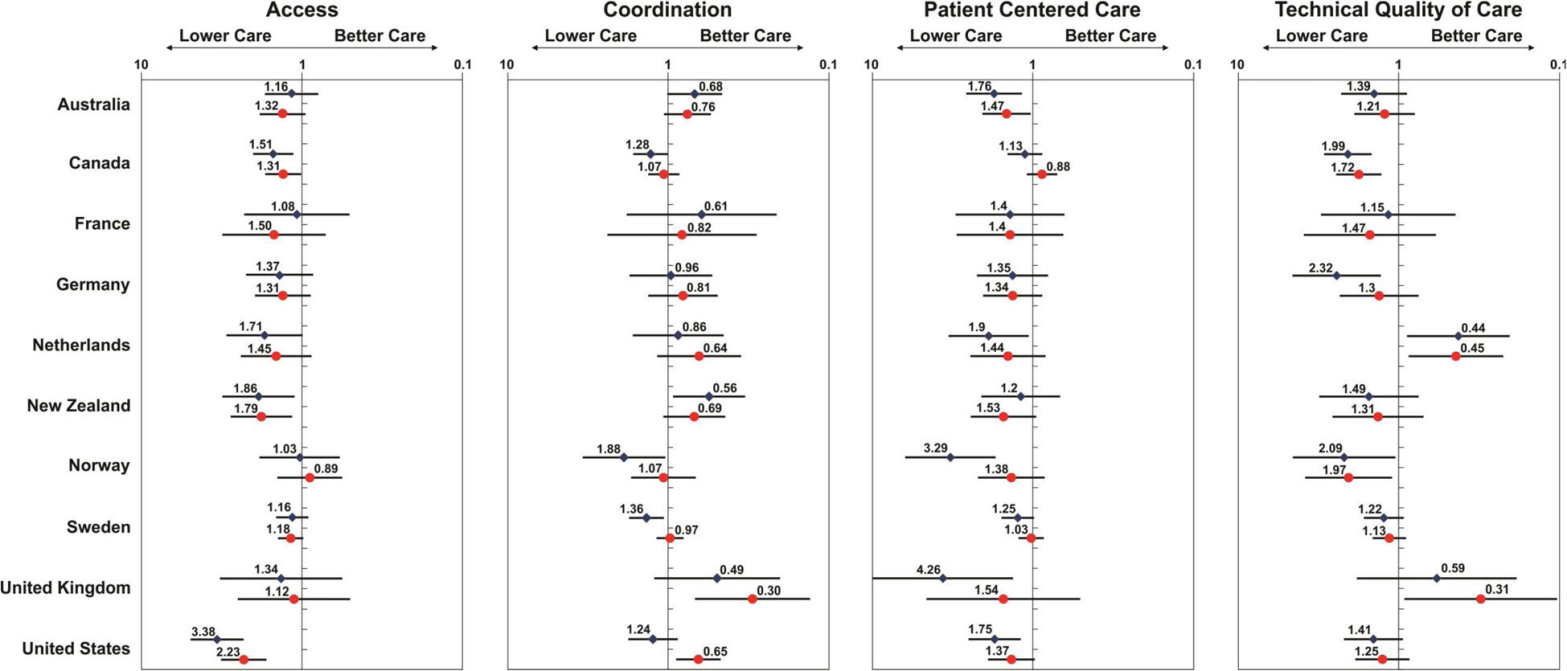
Odds of reporting “poor” care for low and middle income categories relative to upper income category (reference to high income), adjusted for number of individuals in the household. The blue diamond and the red circle represent the odds ratio of individuals living in low income and middle income households, respectively, relative to those in high income households

Questions relating to the performance of the PC system had a high completion rate: 99, 94, 96, and 99% of eligible respondents for access, coordination, patient-centered care, and technical quality of care, respectively. Table 2 shows the proportion of eligible individuals in each income category whose responses indicated “poor” care. Consistently, individuals in the low income category had higher prevalence of “poor” care than those in the higher income group, while those in the middle income category usually had an intermediate score. The spread was highest for technical quality of care; with scores of 53.1, 50.1 and 45.5% reporting “poor” care in the low, middle, and high income group, respectively. Amongst the individuals who did not report their household income, “poor” care was less likely to have been reported than in any other income group for Access: 53.0%; Coordination 56.8; Patient Centered Care: 43.9. Nearly half of these individuals (49.8%) had “poor” Technical quality of care: 49.8%. Table 3 reports the odds ratio of low- and middle-income individuals reporting “poor” care relative to high-income individuals, after adjusting for the number of people living in the household. The results for each country are depicted in Fig. 1. Weighing the records according to their socio-demographic profile to allow population-based estimates, had no impact on the results to two decimal places.

**Table 2:** Percentage of individuals in each income group reporting poor quality of care relative to all eligible respondents in that category

Indicators	Access				Coordination				Patient Centered Care				Technical Quality of Care			
Income	n	Low	Middle	High	n	Low	Middle	High	N	Low	Middle	High	N	Low	Middle	High
Overall	14,143	64.1	63.5	60.2	13,419	63.5	57.5	60.6	13,718	49.5	46.4	45.1	11,008	53.1	50.1	45.5
Sweden	4,149	63.2	64.6	61.7	3679	70.9	62.0	61.3	3,961	62.0	59.0	60.0	3,229	66.7	64.5	61.4
Canada	3,191	79.1	78.4	74.4	3,073	55.7	51.0	49.1	3,061	48.9	43.7	47.2	2,517	44.7	40.6	28.1
Australia	1,236	63.6	68.5	63.2	1,214	59.8	62.8	69.1	1,215	37.0	34.6	27.0	950	48.4	45.2	40.9
United States	964	76.8	70.6	52.2	943	60.1	44.7	55.6	915	43.9	38.6	31.4	826	43.3	40.6	35.4
France	871	42.7	52.3	43.3	862	79.9	83.1	85.7	866	49.2	49.9	41.4	643	65.3	69.3	57.1
Germany	851	57.1	56.9	50.4	849	78.6	76.6	80.5	827	38.2	40.6	34.2	608	51.4	37.8	32.0
United Kingdom	832	44.7	38.7	33.3	808	45.0	29.9	58.3	825	42.7	21.9	16.0	631	35.2	19.3	41.2
Netherlands	741	55.1	52.4	45.9	718	70.7	66.4	78.1	734	45.2	38.6	31.1	596	52.2	52.6	70.6
Norway	671	54.5	53.1	59.6	656	66.4	54.7	55.6	678	81.2	66.3	62.5	554	68.8	67.7	52.5
New Zealand	637	50.0	53.6	41.3	617	39.9	46.5	56.3	636	27.2	35.9	28.2	454	39.6	38.4	32.7

Individuals with incomplete data in the domain studied were excluded (Access = 119, Coordination = 843, Patient Centered Care = 544, Technical Quality of Care =133). These were distributed across all countries. 11,141 were eligible to respond to the technical quality of care questions

**Table 3:**
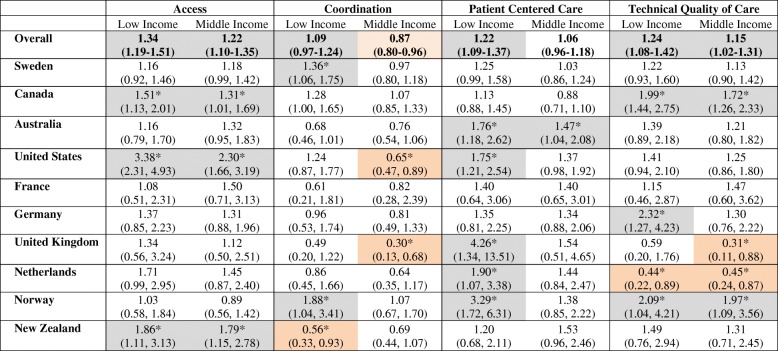
Odds of reporting “poor” care for low and middle income categories relative to upper income category – adjusted for number of individuals in the household

Adjusted for number of individuals in household

Statistically significant results are indicated by a “*”. Within these, results representing worse care for those in the high income group are shaded light orange, whereas those representing worse care for lower income groups are shaded light grey

With one exception (Norway), individuals with low and middle income were more likely to report having had poor access, compared to those with high income. This reached statistical significance for Canada, the United States, and New Zealand. An apparent dose-response relationship was observed where the odds ratio for the lower-income comparison tended to be higher than that of the middle-income comparison.

There was no consistent relationship between income status and the quality of care coordination. Individuals in the lower income group living in Sweden and Norway were more likely to report lower coordination, while in the United Kingdom, New Zealand, and to some extent, the United States, it was individuals in the highest income bracket that reported lower care coordination.

With one exception (Canada), patients in the lower income groups were more likely to report deficiencies in patient centered care. This reached statistical significance in Australia, the United States, the United Kingdom, the Netherlands, and Norway.

Two countries, the United Kingdom and the Netherlands, showed meaningful and statistically significant better measures of technical quality of care for individuals in the lower income bracket. In Canada, Germany and Norway, the effect was in the opposite direction.

## Discussion

### Variation within and across countries

This study is the first to examine the relationship between income inequality and experience of PC services cross-nationally. In our sample of “sicker adults” the main finding was that inequity in primary health care exists in virtually all countries in at least one dimension of care, although there were no within country trends that would point to deficiencies in that healthcare system that compromised equitable care delivery across the four dimensions. Within one country, the association between income status and quality of care sometimes favoured people with greater needs but usually followed the inverse care law in others [25]. The United Kingdom is an interesting example of the extent of variability in performance measure disparity within one country, where we observed the largest gaps in favour of (patient centered care, Odds ratio 4.26, 95% CI [1.34–13.51), and against (technical quality of care, Odds ratio: 0.31 95% CI [0.11–0.88]) individuals in the high income group within a single country. The results for two dimensions, access and patient centered care, point to the presence of a systemic issue affecting many countries.

### Dimensions

#### Access

For virtually all countries, the direction of the association between income and access was in favour of high-income individuals. This reached statistical significance for three countries, where there was also evidence of a dose response association. The odds of reporting poor access increased from high to low income groups, strengthening the notion that the association may be causal [26]. The questions assessing access pertained to the availability and accommodation of PC services and the potential consequence of poor PC access (ER visits). None related to affordability [27]. At the time of this survey, strategies to improve access, such as PC performance benchmarking or PC financial compensation, had been adopted several years prior in Canada, Sweden, Australia, England and the Netherlands [28–30]. The results of this study suggest that these measures did not eliminate gaps in access across income strata in Canada and likely in the Netherland. These results are consistent with a recent systematic review in which no meaningful association was found between reimbursement structure and equitable access to PC across socio-economic groups [31].

#### Patient centered care

We also observed a dose-response pattern favouring high income individuals in patient centered care across most countries; with results achieving statistical significance in five How and why the individual’s socio-economic situation may influence patient-centeredness is unclear. A possible explanation is that the usual encounter time allocation is insufficient to delivering quality Patient-centered Care for individuals with more complex needs [32], and individuals of lower socio-economic status are more likely to have complex needs. Patient centered care is also a complex concept to measure which studies have found to be influenced by patient expectations [33]. Some indicators used to measure patient centered care in this survey (e.g. Does your doctor spend enough time with you?) would likely be influenced by patient expectations. A recent study suggested that socio-demographic conditions contribute to reduced patient centered care mediated through to barriers in access to health information [34].

#### Coordination

There was no consistent trend in equitable delivery of coordinated or technical quality of care across countries. In some countries, individuals in the lower income groups reported better care in these dimensions than their high income counterparts. An analysis of the 2013 survey suggested that care coordination is positively related to encounter time and effective explanation of issues, physician knowledge of the patient’s medical history, and patient engagement in care; [35] aspects of care frequently related to patient centeredness [36, 37]. However, we found no evidence of association between equity in these two measures within countries.

#### Technical quality of care

Three of the six technical quality of care indicators relate to process measures that are usually carried out by the family physician (blood pressure and foot examination, medication discussion) and the extent to which these are done may relate to adequate access to PC services. Three others are not solely tied to the PC service, but require the patient to access additional services (cholesterol, HbA1c and eye checks), which may pose additional access barriers.

### Countries

In Norway, a country with universal public health care, despite having relatively fewer respondents (671) than in other countries, we observed statistically significant inequities across three of four dimensions (Table 3). However, Norway was the only country where there was no evidence of inequity in access to PC [23, 38]. These results are similar to a national survey of Norwegians that found respondents reported equal access to General Practitioners and inpatient hospital care but inequity in access to private specialists and outpatient hospital based care [38]. The authors suggest that income-based inequities at some point during the doctor/patient interaction might explain why they found income-based inequities in the utilization of private medical specialists by Norwegian adults [38]. In a separate study in Norway, the odds of reporting poor technical quality of care were approximately two times higher for lower-income patients versus the high-income patients in this sample which the authors felt could reflect a lack of reforms in Norway’s health system that address a need for increased patient safety in PC [23]. Our data suggest that lower-income patients may be at a greater risk than high-income patients with regard to patient safety. This is especially important findings as our analyses revealed the largest differences in the self-reported health measure in Norway and the United States.

The Canada Health Act states that all Canadians, rich or poor, should have access to the same level of health care once insured through the publically funded health system [39]. Yet, Canadian findings showed significant evidence of income-based inequity in two dimensions; technical access and quality of care. Since Canada has a single-payer universal health system that eliminates access barriers related to service costs, the cause of this finding may lie in other barriers more indirectly related to the individual’s financial situation. A recent phone audit study conducted in Ontario, Canada suggests that discrimination could play a role [40]. In that study, researchers contacted 375 PC Clinics in Toronto, and found that reception staff was more likely to offer an appointment to “patients” (actually the researchers) who they perceived as having a high socio-economic status versus those with a perceived low socio-economic status. Individuals in low income brackets also tend to have less flexibility in their work hours and may require higher scheduling accommodations. Geography and physician availability are most likely implicated as well. Rural areas are more likely to be inhabited by lower-income patients and while approximately 21% of Canadians live in rural areas, only 9.4% of family physicians provide service to rural areas [40]. Future studies should investigate the proportion of participants living in rural areas and assess the effects on ratings of access. Canadian findings also revealed inequities in the technical quality of care.

The US showed the highest gap for access to PC between the lower and higher-income group. This may be unsurprising, given the US health system’s strong orientation toward specialized medicine, private insurance coverage and large population of uninsured citizens. The major discrepancy between the US and other participating countries on access is consistent with the concerns regarding affordability reported in a previous Commonwealth Fund survey [12, 41]. Since the time of the 2011 survey, the structure of the United States’ health care system has undergone a significant shift as a result of the 2010 Affordable Care Act (ACA) (responsible for extending coverage to 22 million people nationally).

New Zealand results also showed significant inequity for access to PC. In other studies New Zealand has been shown to score well on several indicators of access to PC [12, 41]; though Emergency Department (ED) usage rates have been shown to be fairly high comparable to other nations (Canada and the US had the highest rates) [41]; A 2014–2015 report on ED use from the New Zealand Ministry of Health shows that ED usage rates were greater among patients who scored high on socio-economic deprivation [42]. New Zealand recently announced an expansion of their program to provide low-cost access to PC to the poorest in the country, an indication that this is a recognized problem. Further investigation is warranted to better understand these findings and the potential impact of the recent policy decisions. New Zealand and the United Kingdom had fewer individuals reporting problems in care coordination, and these countries showed pro-poor performance in these measures across income groups.

Sweden performed well on income-based equity with the exception of the low-income comparison on coordination. Recent health reforms in Sweden (i.e., the Choice reform in Stockholm County Council in 2008 and the Primary Health Care Choice Reform in 2010) could explain our results for Sweden on this dimension. These reforms were designed to increase access and patient choice by allowing PC providers to launch private for-profit practices, effectively terminating a health system of needs-based resource distribution. It has been suggested that these reforms primarily benefit the socially advantaged and that lower-income patients with complex needs experience difficulty obtaining integrated care [43].

France, Germany and the Netherlands similarly showed little evidence of income-based inequity on our dimensions of PC; however, a very high percentage of participants (> 70%) from these nations reported poor coordination of care, regardless of their income (Table 3). In fact, coordination was rated poorly by the majority of participants in nearly all countries [7].

### Limitations and future directions

The data for this study were collected by the Commonwealth Fund and not originally intended for the purposes this study. There was a large variation in the individual countries’ sample size and consequently statistical power to detect significant differences in the quality measure across income groups. The lack of statistical significance in the presence of an important effect size (e.g. Access in the Netherlands), and statistically significant findings that may not have a strong effect size (e.g. Coordination in Sweden) should be interpreted with caution. We sought to represent the burden of inequity across the individuals living in these countries. We did not seek to account for factors that can contribute or explain away these inequities.

Since there were few consistencies cross-nationally, it is plausible that macro-level health system policy or system configuration within each country could explain our results, including intra-country variation across dimensions.. Future work should examine the relationship between inequity in primary health care and health system factors, like physician remuneration and practice structure, health insurance design [44] and the implementation of specific PC reforms [43].

## Conclusion

The findings presented in this paper broaden our understanding of the relationship between income and PC performance across the access, care coordination, patient centered care, and technical quality of care dimensions. It demonstrates that income related inequities in at least one dimension of care exist for nearly all participating OECD countries. While PC has been demonstrated to be an essential component of the health care system in promoting equitable access to health resources and reducing disparities in health across population strata, this study shows that income-based inequities are present within the walls of PC. It is essential that researchers and policy makers seek to uncover what makes a PC system more equitable to mitigate barriers to making quality PC accessible to all.

## Additional file

Additional file 1: Appendix A. (DOCX 30 kb)
