# Supplementary material for: Equity of primary care service delivery for low income “sicker” adults across 10 OECD countries
Source: Int J Equity Health. 2018 Dec 12;17:182. doi: 10.1186/s12939-018-0892-z (PMC6292158; doi:10.1186/s12939-018-0892-z)
Supplement: Supplementary file 1 — Appendix A. (DOCX 30 kb) [file 12939_2018_892_MOESM1_ESM.docx]

# Appendix A

## EXPOSURE

| **Indicator** | **Survey Item(s)** |
| --- | --- |
| Income | Qs2010and 2020. Was your household income…?   1. Less than $34,000 2. $34,000 to less than $62,000 3. $62,000 to less than $76,000 4. $76,000 to less than $105,000 5. $105,000 to less than $140,000   $140,000 or more |
| Number people in household | Q1735. Including you, how many adults, 18 or older, live in your household?  # |
|  | Q1740. How many children under the age of 18 live in your household?  # |

## SOCIO-DEMOGRAPHIC PROFILE*

| Age | Q710a1. What is your year of birth?  DOB |
| --- | --- |
| Gender | Q720. Are you…?   1. Male 2. Female |
| Education | Q1755. What is the highest level of education you have completed to date?   - 1. Less than high school   2. Some high school   3. High school graduate or equivalent   4. Some community college, technical, trade, or vocational college   5. Community college degree or diploma   6. Some university, but no degree   7. University degree or higher - Not sure - Decline to answer |
| Immigration | Q1705. Were you born in Canada or somewhere else?   1. Yes, born in this country 2. No, born somewhere else    - Not sure  - Decline to answer |
| Years lived in Canada | Q1710. At what age did you enter Canada?  # |

*See IHP 2011 Methods Report.doc by CWF for details on population weights for indicators.

## MORBIDITY

| Health description | Q725. In general, how would you describe your own health?   - - 1. Excellent     2. Very good     3. Good     4. Fair     5. Poor     - Not sure     - Decline to answer |
| --- | --- |
| Chronic conditions | Q1416 (A1-A8). Do you have…?   - 1. Hypertension (sometimes called high blood pressure)   2. Heart disease, including angina or heart attack   3. Diabetes   4. Joint pain or arthritis   5. Asthma, COPD, or any other chronic lung problems   6. Depression, anxiety or other mental health problems   7. Cancer   8. Chronic back pain   9. Yes   10. No   - Not sure - Decline to answer |

## PRIMARY CARE PERFORMANCE

|  |  |
| --- | --- |

| **Access** | Regular doctor | Q905. Is there one doctor you usually go to for your medical care?   1. Yes, have a regular doctor/GP 2. Yes, but have more than one regular doctor/GP 3. No    - Not sure  - Decline to answer |
| --- | --- | --- |
|  | Out of hours access | Q815. Last time when you needed medical care in the evening, on a weekend or on a holiday, how easy or difficult was it to get care without going to the hospital emergency room?  1 Very easy  2 Easy  3 Somewhat difficult  4 Very difficult  5 Never needed care in the evenings, weekends, or holidays   - Decline to answer |
|  | Time to see doctor | Q820. Last time you were sick, how quickly could you get to see a doctor or nurse?  Please do not include a visit to the hospital emergency room.  1 On the same day  2 The next day  3 In 2 to 3 days  4 In 4 to 5 days  5 In 6 to 7 days  6 After more than a week  7 Never able to get an appointment/consultation   - - Not sure - Decline to answer |
|  | Answer to questions | Q935. When you call your regular doctor’s office with a medical question or concern during regular practice hours, how often do you get an answer that … day?  1 Always  2 Often  3 Sometimes  4 Rarely or never  5 Never tried to contact by telephone   - Decline to answer |
|  | Reason for ER use | Q1410. The last time you went to the hospital emergency room, was it for a condition that you thought could have been treated by your regular doctor if he or she had been available?  1 Yes  2 No   - Not sure - Decline to answer |
| **Coordination** | Coordination with specialist | Q1050. When you saw the specialist did he or she have information about your medical history?   1. Yes 2. No  - Not sure - Decline to answer |
|  | Coordination for patient | Q1060. How often does your regular doctor or someone in your doctor’s practice help coordinate or arrange the care you receive from other doctors and places, such as make appointments?  1 Always  2 Often  3 Sometimes  4 Rarely or never  5 I do not receive care from other doctors or places   - Not sure - Declined to answer |
|  | Doctor(s) for chronic condition | Q1462. Is there one person responsible for all the care you receive from various doctors for your chronic condition(s)?  1 Yes  2 No   - Not sure - Decline to answer |
| **Patient- Centered Care** | Treatment from regular doctor | Q941 (A1-A4). When you receive care or treatment, how often does your regular doctor or someone in your doctor’s practice…?  Would you say always, often, sometimes, rarely or never?   - 1. Know important information about your medical history   2. Spend enough time with you   3. Encourage you to ask questions   4. Explains things in a way that is easy to understand   5. Always   6. Often   7. Sometimes   8. Rarely or never - Not applicable - Declined to answer |
| **Technical Quality of Care** | Discussion of medications | Q1110. In the past year, has a pharmacist or any doctor reviewed and discussed … the different medicines you are using?  1 Yes  2 No   - Not sure - Decline to answer |
|  | Cholesterol check | Q1420. Have you had your cholesterol checked in the past year?   1. Yes 2. No  - Not sure - Decline to answer |
|  | Blood pressure check | Q1425. Have you had your blood pressure checked in the past year?  1 Yes  2 No   - Not sure - Decline to answer |
|  | HbA1c check | Q1435. Has your hemoglobin “A one C,” a blood test to check sugar control, been checked in the past year?  1 Yes  2 No   - Not sure - Decline to answer |
|  | Diabetes complications | Q1446 (A1-A2). Has your…?   - 1. Your feet examined by a health professional for sores or irritations in the past year   2. An eye examination for your diabetes in the past year   1 Yes  2 No   - Not sure - Decline to answer |
